# 1070. *In vitro* Activity of PLG0206 Against Isolates Commonly Found in Periprosthetic Joint Infections (PJI)

**DOI:** 10.1093/ofid/ofab466.1264

**Published:** 2021-12-04

**Authors:** David Huang, Jonathan Steckbeck, Chris Pillar, Bev Murray, David Huganfel, Dean Shinabanger

**Affiliations:** 1 Peptilogics, Houston, Texas; 2 Micromyx, Kalamazoo, Michigan

## Abstract

**Background:**

PLG0206 is a novel engineered cationic antimicrobial peptide being evaluated for treatment of prosthetic joint infections. In this study, the activity of PLG0206 was evaluated by broth microdilution against 104 isolates of *Staphylococcus epidermidis*, 53 other coagulase-negative staphylococci (CoNS), 3 *S. aureus,* and 66 Gram-negative isolates consisting of Enterobacterales, *Pseudomonas aeruginosa,* and *Acinetobacter baumannii*.

**Methods:**

Imipenem, levofloxacin, tigecycline, linezolid, vancomycin, oxacillin, ceftazidime, colistin, and amikacin were tested as comparators. Testing was conducted in accordance with guidelines from the Clinical and Laboratory Standards Institute (CLSI; M7 and M100). Test organisms consisted of reference strains from the American Type Culture Collection, the Centers for Disease Control Antibiotic Reference Bank and clinical isolates from the Micromyx repository. The media employed for testing in the broth microdilution MIC assay for all organisms were cation-adjusted Mueller Hinton Broth and for PLG0206 only included RPMI-1640 medium supplemented with 0.002% P-80.

**Results:**

Activity of PLG0206 in RPMI against CoNS, *S. aureus,* and resistant Gram-negative* pathogens are shown in Table.

Activity of PLG0206 in RPMI against CoNS, S. aureus and resistant Gram-negative* pathogens

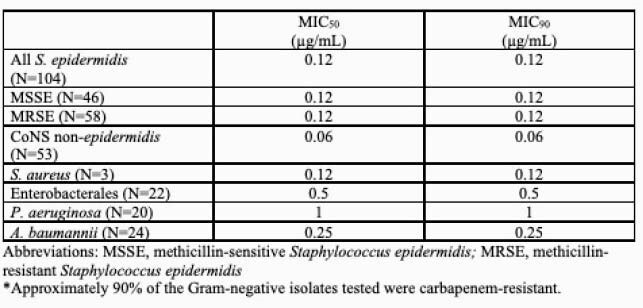

Activity of PLG0206 in RPMI against CoNS, S. aureus and resistant Gram-negative* pathogens

**Conclusion:**

PLG0206 was found to have potent antimicrobial activity when evaluated in RPMI against

*S. epidermidis*, CoNS non-epidermidis, *S. aureus*, Enterobacterales, *P. aeruginosa*, and *A. baumannii,* including isolates with multi-drug resistance.

**Disclosures:**

**David Huang, MD, PhD**, **Peptilogics** (Employee) **Jonathan Steckbeck, PhD**, **Peptilogics** (Employee) **Chris Pillar, PhD**, **Micromyx** (Employee) **Bev Murray, BS**, **Micromyx** (Employee) **David Huganfel, BS**, **Micromyx** (Employee) **Dean Shinabanger, PhD**, **Micromyx** (Employee)

